# Three Novel Mutations in the* NPHS1* Gene in Vietnamese Patients with Congenital Nephrotic Syndrome

**DOI:** 10.1155/2017/2357282

**Published:** 2017-03-14

**Authors:** Thi Kim Lien Nguyen, Van Dem Pham, Thu Huong Nguyen, Trung Kien Pham, Thi Quynh Huong Nguyen, Huy Hoang Nguyen

**Affiliations:** ^1^Institute of Genome Research, Vietnam Academy of Science and Technology, Hanoi, Vietnam; ^2^Faculty of Medicine and Pharmacy, Vietnam National University, Hanoi, Vietnam; ^3^Vietnam National Hospital of Pediatrics, Hanoi, Vietnam; ^4^Hanoi Medical University, Hanoi, Vietnam

## Abstract

Congenital nephrotic syndrome, a rare and severe disease, is inherited as an autosomal recessive trait. The disease manifests shortly after birth and occurs predominantly in families of Finnish origin but has now been observed in all countries and races. Mutations in the* NPHS1* gene, which encodes nephrin, are the main causes of congenital nephrotic syndrome in patients. In this study, we report the first mutational analysis of the* NPHS1* gene in three unrelated children from three different Vietnamese families. These patients were examined and determined to be suffering from congenital nephrotic syndrome in the Department of Pediatrics, Vietnam National Hospital of Pediatrics. All 29 exons and exon-intron boundaries of* NPHS1* were analyzed by PCR and DNA sequencing. Genetic analysis of the* NPHS1* gene revealed one compound heterozygous variant p.Glu117Lys, one heterozygous missense mutation p.Asp310Asn, and one heterozygous frame-shifting mutation (c.3250_3251insG causing p.Val1084Glyfs⁎12) in patient 1. In patient 2, one heterozygous variant p.Glu117Lys and one novel heterozygous missense mutation p.Ser324Ala were identified. Finally, a novel missense mutation p.Arg802Leu and a novel nonsense mutation (c.2442C>G causing p.K792⁎) were identified in patient 3.

## 1. Introduction

Congenital nephrotic syndrome (CNS) is a rare disease that has been defined as the occurrence of nephrotic syndrome (NS) within the first 3 months of life. CNS is inherited as an autosomal recessive trait. CNS is a life-threatening kidney disorder clinically characterized by excessive proteinuria and edema. The most common form of the disease is Finnish CNS (CNF, congenital nephrotic syndrome of the Finnish type), and now CNS (non-Finnish type) has been observed in all countries and races. Congenital nephrotic syndrome is an inherited disorder caused by mutations in the* NPHS1* gene. The* NPHS1* gene (OMIM *∗*602716) has been mapped to chromosome 19q13.1 and consists of 29 exons that span a 150kb region.* NPHS1* encodes the 1241 amino acid nephrin protein [1], the single most important component of the slit diaphragm [2].

Nephrin is a transmembrane glycoprotein that belongs to the immunoglobulin (Ig) superfamily [1, 3, 4]. Nephrin participates in the structural basis of the slit diaphragm that consists of eight extracellular Ig-like domains, one fibronectin type III motif, a short transmembrane region, and a cytosolic C-terminal tail [5]. Based on the structure, it is hypothesized that nephrin molecules from adjacent foot processes interact in the center of the filtration slit diaphragm to form a zipper-like structure that is too small for albumin-sized molecules to pass [3, 5]. Nephrin plays a crucial role in the selective filtration function of the slit diaphragm, as massive proteinuria is a consequence of nephrin absence or malfunction [6]. Recent studies have indicated that nephrin homotypic interactions influence cytoplasmic posttranslational modifications and signaling [3, 7, 8].

More than 220 mutations have been described in the* NPHS1* gene; the majority of these mutations were truncation and missense mutations. Missense mutations accounted for more than 50% of extracellular domain mutations and 66% of these occur in Ig domains leading to mutational hot spots. Missense mutations of the* NPHS1* gene lead to the abnormal retention of nephrin in the endoplasmic reticulum, therefore failing to traffic out to the cell surface [9]. Koziell et al. [10] reported that most mutations causing CNS with a severe clinical phenotype were observed in Ig2, Ig4, and Ig7 of nephrin. Mutations of the* NPHS1* gene can lead to disruption of the filtration barrier and are related to the early onset of disease. Milder cases resulting from mutant* NPHS1* had either two mutations in the cytoplasmic tail or two missense mutations in the extracellular domain, including at least one that preserved structure and function [11].

The* NPHS1* mutation detection rate approaches 98% in children with CNS in Finland [1] with the most prevalent ones reported in exons 2 (p.Leu41fs*∗*90) and 26 (p.Arg1109*∗*), which are referred to as Fin-major and Fin-minor, respectively [12–14]. However, the rate of mutation of the* NPHS1* gene varies amongst different ethnic groups outside of Finland ranging from 39 to 80% in CNS cases [15–19]. Mutations in the* NPHS1* gene in the Japanese and Chinese probands diagnosed with CNS have also been reported [20–22].

To the best of our knowledge, no mutation in the* NPHS1* gene has been published for a Vietnamese CNS cohort. Therefore, we aimed to detect known and novel mutations in the* NPHS1* gene of three unrelated children with CNS from three different Vietnamese families.

## 2. Case Presentation

### 2.1. Patient 1

A 40-day-old boy was admitted in the Department of Pediatrics, Vietnam National Hospital of Pediatrics. He was a full-term normal delivery with a birth weight of 2.8 kg. The weight of the placenta was unknown. The biochemical indices of the blood serum revealed 27.2 g/L serum total protein (normal is > 56 g/L), 8.84 g/L albumin (normal is > 25 g/L), and 10.9 mM/L cholesterol. The biochemical indices of the urine revealed 6,100 mg/L protein (normal is < 200 mg/L) and 8,918 mg/L protein/creatinine (normal is < 300 mg/L). Patient had a whole-body edema, multimembrane effusion, severe pneumonia, severe decrease blood protein and plasma albumin, and high levels of protein in urine, recurrent many times. Patient was diagnosed with congenital nephrotic syndrome. His parents had normal urinalysis, but his older brother was also diagnosed with congenital nephrotic syndrome and died at sixth month by renal disease. However, we did not collect a sample of his brother's DNA for genetic analysis.

### 2.2. Patient 2

A 2-month-old boy was a full-term normal delivery with a birth weight of 2.8 kg. The weight of the placenta was unknown. The biochemical indices of the blood serum revealed 35.3 g/L serum total protein (normal is > 56 g/L), 10.7 g/L albumin (normal is > 25 g/L), and 7.91 mM/L cholesterol. The biochemical indices of the urine revealed 11,600 mg/L protein (normal is < 200 mg/L) and 12,070 mg/L protein/creatinine (normal is < 300 mg/L). Patient was diagnosed with congenital nephrotic syndrome. Patient required admission twice to Vietnam National Hospital of Pediatrics for anasarca during the first two months of life but has done well since.

### 2.3. Patient 3

A 2.5-month-old boy was a full-term normal delivery with a birth weight of 3.2 kg. The weight of the placenta was unknown. The biochemical indices of the blood serum revealed 28 g/L serum total protein (normal is > 56 g/L), 9.5 g/L albumin (normal is > 25 g/L), and 12.1 mM/L cholesterol. The biochemical indices of the urine revealed 22,300 mg/L protein (normal is < 200 mg/L) and 11,262 mg/L protein/creatinine (normal is < 300 mg/L). Patient was diagnosed with congenital nephrotic syndrome. Patient had a severe pneumonia, diarrhea and severe dehydration, kidney failure, and rapid development to end-stage renal failure and died. His parents had normal urinalysis, but his older brother was also diagnosed with congenital nephrotic syndrome and died at tenth month by renal disease. We also did not collect a sample of his brother's DNA for genetic analysis. However, their parents and older sister are healthy with negative proteinuria and hematuria; other indicators such as blood protein, albumin, creatinine, and urea are normal. The sister's kidneys were normal on ultrasound evaluation.

The patients were examined and determined to be suffering from congenital nephrotic syndrome in the Department of Pediatrics, Vietnam National Hospital of Pediatrics in 2015.

### 2.4. Genetic Analysis

Genomic DNA was isolated from blood samples (including of the patients and their families: father, mother, and older sister of patient 3) using a GeneJET Genomic DNA purification kit (Thermo, USA) and following manufacturer guidelines. The DNA concentration was determined using a Thermo Scientific NanoDrop spectrophotometer (Waltham, MA, USA).

The primers for amplifying exons 1–29 were synthesized according to published information regarding intron-exon boundaries [15]. All synthetic oligonucleotide primers were synthesized and purchased from IDT (USA). Fifty nanograms of genomic DNA was subjected to 35 cycles of PCR amplification in a 25 *μ*L volume consisting of 1 *μ*L 5 pM sense primer, 1 *μ*L 5 pM antisense primer, 1.5–3.5 mM MgCl_2_, 100 *μ*M dNTPs, and 1.25–1.5 U DreamTaq polymerase (Thermo, USA). DNA was denatured at 95°C for 12 min followed by 35 cycles of denaturation for 1 min at 95°C, annealing for 1 min at 60–65°C, and extension for 1 min at 72°C, and a final extension for 7 min at 72°C. The PCR amplification was carried out on an Eppendorf Mastercycler EP gradient (USA Scientific, Inc).

DNA sequencing was performed in both directions, initiated from the forward and the reverse primers, which had been used in the initial PCR reaction. PCR products were sequenced by method of direct sequencing on ABI 3100 Bio System (USA). The sequencing data were analyzed and compared against the reference* NPHS1* gene sequence published in Ensembl (ENST00000378910) by using BioEdit software to determine the nucleotide changes.

### 2.5. In Silico Analysis

The consequences of any novel nonsynonymous nucleotide variations that were identified within exons were evaluated with the in silico analysis tools sorting intolerant from tolerant (SIFT) prediction [23], polyphen 2 [24], and mutation taster [25].

### 2.6. Amino Acid Conservation

The amino acid sequences of NPHS1 from different species including green monkey (*Chlorocebus sabaeus*, XM007996435), Nancy Ma's monkey (*Aotus nancymaae*, XM012437656), human (*Homo sapiens*, NM004646), Rhesus monkey (*Macaca mulatta*, XM015123713), yak (*Bos mutus*, XM005908211), sheep (*Ovis aries*, XM012190201), Przewalski's horse (*Equus przewalskii*, XM008543338), cheetah (*Acinonyx jubatus*, XM015072043), western gorilla (*Gorilla gorilla*, XM004060548), alpaca (*Vicugna pacos*, XM006217092), and house mouse (*Mus musculus*, AF191090) were aligned using Clustal W to determine the evolutionary conservation of old type amino acid residues at the position of substitutions.

## 3. Discussion

### 3.1. Patient 1

**Figure 1:**
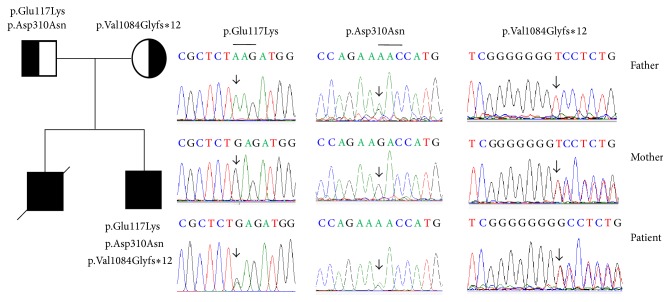
Mutations of the* NPHS1* gene were identified by sequencing in patient 1 and his parents. The pedigree and mutations of the* NPHS1* gene were identified in the family of patient 1, including a heterozygous variant p.Glu117Lys, a mutation p.Asp310Asn, and a heterozygous frameshift mutation p.Val1084Glyfs*∗*12.

A heterozygous polymorphism in exon 3 of* NPHS1*, c.349G>A (p.Glu117Lys), was identified in patient 1. Genetic analysis of the patient's parents showed that the father had a homozygous adenine (A) at position 349 in cDNA and the mother has a homozygous guanine (G) at position 349 in cDNA. Patient 1 also had a heterozygous missense mutation, c.928G>A (p.Asp310Asn), in exon 8 of* NPHS1*. The child's father had normal urinalysis and the same heterozygous missense mutation c.928G>A (p.Asp310Asn); however, the patient's mother had a wildtype G at position 928 in cDNA. A heterozygous mutation in exon 24 of* NPHS1*, c.3250_3251insG, leading to a frameshift mutation p.Val1084Glyfs*∗*12, was also identified in patient 1 (Table 1). Further mutational analysis of the* NPHS1* gene in the parents of patient 1 showed a heterozygous mutation, c.3250_3251insG, in the mother (Figure 1).

The p.Glu117Lys mutation was reported by Lenkkeri et al. [15] as a single nucleotide polymorphism in a CNS cohort and has now been accepted as a well-known polymorphism.

The c.928G>A mutation within exon 8 has been published in previous studies [20, 26]. This missense mutation was predicted to lead to the replacement of an aspartic acid by an asparagine at position 310 in Ig3 in the nephrin protein and is accepted as a pathological mutation [20]. Fu et al. [26] reported a CNS patient that had two heterozygous single-base mutations (c.928G>A and c.1440+1G>A) in the* NPHS1* gene. However, the patient's mother had a normal phenotype with a heterozygous mutation c.928G>A. The father of patient 1 had a normal phenotype with a heterozygous mutation p.Asp310Asn, and a heterozygous variant p.Glu117Lys; these results were consistent with findings reported by Fu et al. [26].

The* NPHS1* mutation c.3250_3251insG, which leads to a frameshift and a truncated nephrin protein p.Val1084fs*∗*1095, was previously reported and accepted as a pathological mutation [15, 27, 28]. Santín et al. [28] considered that nonsense and frameshift mutations, which are predicted to result in a truncated protein, are classified as severe mutations. The homozygous mutation c.3250_3251insG was found in a Chinese patient with hypoalbuminemia and a serum albumin level of 4.6 g/L 6 days after birth, suggesting that these severe mutations can cause a corresponding severe clinical phenotype [29]. Liu et al. [9] studied the subcellular localization of the mutants in transfected human embryonic kidney cells (HEK293). Their results showed that misfolding and defective intracellular transport was the most common cause in development of the nephrotic syndrome in patients carrying missense mutations in* NPHS1*.

Previous reports suggest that insertion, deletion, and nonsense mutations lead to synthesis of a premature polypeptide, whereas missense mutations lead to a fully mature protein but with amino acid substitutions [12, 15]. Investigating missense mutations in the nephrin gene provided a valuable insight into the pathogenesis of the disease. These mutations affect the extracellular, transmembrane, and cytosolic regions of the nephrin protein. However, it is still unknown how the different missense mutations affect the nephrin protein and cause the same severe disease [15]. Machuca et al. [11] showed that 54% of CNS cases had two heterozygous mutations and 2.5% had a compound of a heterozygous mutation and a nonsilent variant in the nephrin gene. In our study, the severe phenotype observed in patient 1 may be explained by the simultaneous presence of two heterozygous mutations and one heterozygous variant.

### 3.2. Patient 2

**Figure 2:**
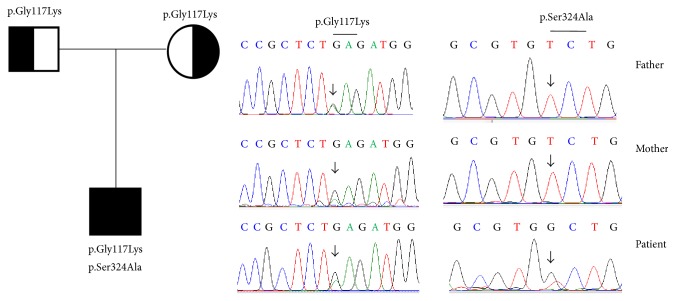
Mutations of the* NPHS1* gene were identified by sequencing in patient 2 and his parents. The pedigree and mutations of the* NPHS1* gene were identified in the family of patient 2, including a heterozygous variant p.Glu117Lys and a “de novo” heterozygous mutation p.Ser324Ala.

The G>A transition at position 349 in exon 3 of* NPHS1* was found to be a p.Glu117Lys amino acid change in patient 2. This nucleotide change was also found in both parents. The T>G nucleotide change was detected at position 970 of exon 8 in cDNA. This “de novo” mutation led to a p.Ser324Ala amino acid change (Figure 2) and was not found in the parents (Table 1). This mutation was not recorded in the NCBI (dbSNP), Ensembl SNPs, 1000 Genomes Project (TGB), the Human Gene Mutation Database (public HGMD), ClinVar, The Exome Aggregation Consortium (ExAC) browser, or NHLBI Exome Sequencing Project (ESP) server databases.

While patient 2 had a mutation leading to a change of serine to alanine in Ig2, a mild disease phenotype was observed. This mild phenotype may be explained by two mutations affecting the structure and function of the protein; however, the resulting changes were not serious. Other milder cases, reported by Machuca et al. [11], had two missense mutations in the extracellular domain, including at least one that preserved structure and function of NPHS1.

PolyPhen-2 analysis [24] for the Ser324Ala substitution in NPHS1 of patient 2 pointed to a “possibly damaging” status with a score of 0.927. The mutation taster tool [25] predicted that a Ser324Ala mutation may cause the occurrence of disease. Analysis performed by SIFT [23] resulted in a score of 0.23 that indicates that the substitution is “tolerated” and has no effect on protein structure. The parents of patient 2 are healthy and do not have any symptoms or abnormal kidney function. This result may be due to a mutation arising in the patient during the formation of gametes or during pregnancy. The rate of novel mutation detection arising in the offspring that were not detected in the parent is reported to be approximately 20% of all mutations detected in the children that were not originally identified in the parents [11, 16].

### 3.3. Patient 3

**Figure 3:**
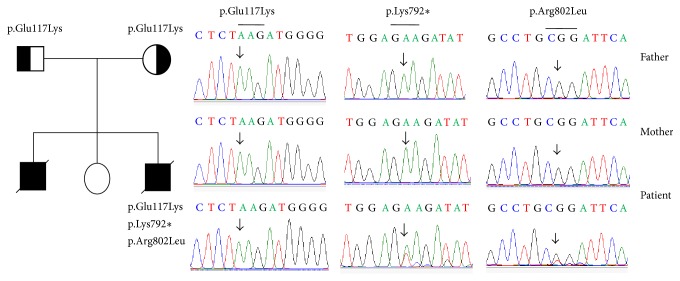
Mutations of the* NPHS1* gene were identified by sequencing in patient 3 and his parents. The pedigree and mutations of the* NPHS1* gene were identified in the family of patient 3, including of a homozygous variant p.Glu117Lys, a heterozygous mutation p.Lys792*∗*, and a heterozygous mutation p.Arg802Leu.

In patient 3, two novel heterozygous mutations were found in exon 18 of the* NPHS1* gene. One mutation was an A>T nucleotide change at position 2374 in cDNA, leading to a nonsense mutation p.Lys792*∗*. One other mutation was a G>T nucleotide change at position 2405 in cDNA, leading to a p.Arg802Leu mutation. These nucleotide changes were not found in the parents (Figure 3). Patient 3 was simultaneously carrying a homozygous variant p.Glu117Lys. This homozygous variant p.Glu117Lys was also observed in both parents (Table 1).

Nucleotide changes at position 2405 in cDNA were reported by Lenkkeri et al. [15]; these include a nucleotide change G>C at position 2405 in cDNA, which led to an amino acid substitution p.Arg802Pro, and a nucleotide change C>T at position 2404 in cDNA, which led to an amino acid substitution p.Arg802Trp. In our study, a nucleotide change G>T led to amino acid substitution p.Arg802Leu, indicating a novel mutation in a Vietnamese patient with CNS.

Interestingly, this patient also had a heterozygous mutation p.Lys792*∗*, which is also a novel mutation. However, both mutations in patient 3 were not identified in either the father or the mother. It is also noted that the patient's older brother died because of CNS, whereas the elder sister was healthy. According to Liu et al. [9], mutations leading to single amino acid substitutions essentially caused the same severe phenotype. Some of these mutations have been suggested as a possible cause of misfolding of the mutant protein and therefore may affect correct intracellular transportation [30]. The crystal molecular structure of nephrin has not yet been determined; however, some nonconservative amino acid substitutions such as p.Trp64Ser, p.Ile171Asn, p.Ser350Pro, p.Arg802Trp, and p.Arg802Pro may lead to incorrect conformations in their respective domains. Some reports have shown that protein misfolding due to missense mutations is a common mechanism in the pathogenesis of several human diseases [31, 32].

**Figure 4:**
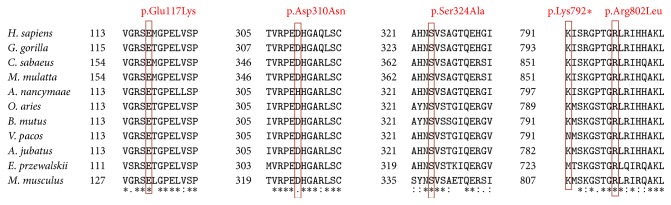
Alignment of amino acid sequences of NPHS1 from different species such as* Chlorocebus sabaeus* (XM007996435),* Aotus nancymaae* (XM012437656),* Homo sapiens* (NM004646),* Macaca mulatta* (XM015123713),* Bos mutus* (XM005908211),* Ovis aries* (XM012190201),* Equus przewalskii* (XM008543338),* Acinonyx jubatus* (XM015072043),* Gorilla gorilla* (XM004060548),* Vicugna pacos* (XM006217092), and* Mus musculus* (AF191090). The positions of the changed amino acids (p.Glu117Lys, p.Asp310Asn, p.Ser324Ala, p.Lys792*∗*, and p.Arg802Leu) in protein NPHS1.

Amino acid sequences of NPHS1 from different species were compared to identify any areas of conservation. Comparison of amino acid sequences (Figure 4) showed that all of the changed amino acids (p.Glu117Lys, p.Asp310Asn, p.Ser324Ala, p.Lys792*∗*, and p.Arg802Leu) were in conservative positions amongst the different species. Although there are no studies on the role of these amino acids, we have observed that changes to amino acids in these positions can influence the function of protein NPHS1 and this may be the cause of CNS in our patients.

In our study, we identified six nucleotide changes in the* NPHS1* gene in three patients with CNS from three unrelated Vietnamese families. Three of the mutations are novel (leading substitution p.Ser324Ala, p.Lys792*∗*, and p.Arg802Leu) in Vietnamese patients. These are considered to be “de novo” mutations, as they were not found in the patient's parents. Our findings demonstrate that inheritance of alleles may contribute to different disease phenotypes. To our knowledge, this is the first study to describe clinical traits and mutations in the* NPHS1* gene in Vietnamese CNS patients. Our findings broaden the known mutation spectrum in CNS patients and will lead to a better understanding of CNS in different ethnic groups beyond the Finnish cohort.

## Figures and Tables

**Table 1:** *NPHS1* mutations detected in the Vietnamese patients with CNS.

Patient	Exon	Nucleotide exchange	Effect on coding sequence	Mutation status	Segregation	Reference
1	3	c. 349G>A	p.Glu117Lys	het	p	[15]
	8	c. 928G>A	p.Asp310Asn	het	p	[20]
	24	c.3250_3251insG	p.Val1084Glyfs*∗*12	het	m	[1]

2	3	c. 349G>A	p.Glu117Lys	het	pm	[15]
	8	c. 970T>G	p.Ser324Ala	het	“De novo”	Present study

3	3	c. 349G>A	p.Glu117Lys	homo	p, m	[15]
	18	c. 2374A>T	p.Lys792*∗*	het	“De novo”	Present study
	18	c. 2405G>T	p.Arg802Leu	het	“De novo”	Present study

(het: heterozygous; homo: homozygous; p: paternal; m: maternal).

## Abbreviations

CNF: Congenital nephrotic syndrome of the Finnish type
CNS: Congenital nephrotic syndrome
cDNA: Complementary DNA
DNA: Deoxyribonucleic acid
ExAC: The exome aggregation consortium
HGMD: Human gene mutation database
NCBI: National center for biotechnology information
NS: Nephrotic syndrome
OMIM: Online Mendelian inheritance in man
PCR: Polymerase chain reaction
SIFT: Sorting intolerant from tolerant
SNP: Single nucleotide polymorphism.
